# The need to be alert to complications of peri‐lead cerebral edema caused by deep brain stimulation implantation: A systematic literature review and meta‐analysis study

**DOI:** 10.1111/cns.13802

**Published:** 2022-01-19

**Authors:** Yu Tian, Jiaming Wang, Lei Jiang, Zhaohai Feng, Xin Shi, Yujun Hao

**Affiliations:** ^1^ Neurosurgery Department The First Affiliated Hospital of Xinjiang Medical University Urumqi Xinjiang China

**Keywords:** brain edema, complications, deep brain stimulation, peri‐lead

## Abstract

**Background:**

The compatibility of deep brain stimulation (DBS) hardware and MRI scans has greatly improved the diagnostic rate of postoperative peri‐lead edema (PLE). However, the etiology, incidence, and prognostic outcomes of this complication have not been established.

**Objective:**

The incidence of PLE and associated symptoms, the process of occurrence and progression of this complication, as well as treatment strategies were evaluated.

**Methods:**

We conducted a Preferred Reporting Items for Systematic Reviews and Meta‐Analyses compliant systematic review of all studies that reported on incidences of PLE and associated symptoms after DBS implantation. Through systematic literature review, we evaluated its causes, neuropsychiatric symptoms, duration, treatment methods, and prognostic outcomes.

**Results:**

Our search retrieved 10 articles, including 5 articles on PLE and 10 articles on symptomatic PLE. The incidence of PLE was 35.8% (95% CI: 17.0%–54.6%), while the incidence of symptomatic PLE was 3.1% (95% CI: 1.5%–4.7%) accounting for 8.7% of PLE.

**Conclusions:**

This complication is not as rare as previously reported. Therefore, it requires significant attention after DBS implantation. The correlation between its causes, duration, symptoms, and the area involved in edema should be assessed in long‐term prospective clinical studies with large sample sizes.

## Graphical abstract

Previously, peri‐lead edema (PLE) after deep brain stimulation (DBS) operation was considered a rare complication and therefore was not taken seriously. Currently, PLE was reported as a common DBS complication of delayed onset. These two very different views arouse our attention, and we conduct a systematic literature review and meta‐analysis study based on different viewpoints of this complication in different medical centers with the aim of clarifying the occurrence and development of this complication and identifying appropriate treatment strategies, so as to raise the academic attention to this complication.

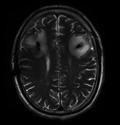

## 1 INTRODUCTION

Deep brain stimulation (DBS) is a surgical procedure in which electrodes or leads that generate electrical impulses targeted towards specific brain regions are implanted at specific locations in the brain. A brain pacemaker initiates the electrical stimulation for the treatment of movement disorders or neuropsychiatric disorders. The DBS surgery is not associated with major structural damages to the brain. However, several complications can occur during clinical treatment.^1^ The main complications include hardware‐related complications (such as lead malposition or migration, component fracture, component malfunction, subcutaneous pocket infection, or allergic reactions), operation‐related complications (such as intracranial hemorrhage, malposition of the electrode, infections, and cerebrospinal fluid leakage), and stimulation‐related complications (such as diplopia, dyskinesia, paresthesia, muscle spasm, and dysarthria).^1^

Previously, peri‐lead edema (PLE) was considered a rare postoperative complication of DBS. Therefore, much attention was not paid to PLE. Increased compatibility of the DBS hardware with MRI scanning has significantly improved the detection rate of postoperative PLE. Currently, PLE is reported as a common DBS complication of delayed onset.

Current studies do not support PLE as an infectious process and speculated to be related to (1) mechanical damage to the punctures lead or accumulation of cerebrospinal fluid (CSF) to electrode puncture needle tract: the blood–brain barrier (BBB) can be damaged by a needle‐stab injury or the transplantation of electrodes, activation of the inflammatory cells can lead to the production and secretion of cytokines, and the cytokines further aggravate the inflammatory process and angiogenesis within hours of the insult.^2^ (2) Venous infarction: neurological deficits that occur shortly after the opening of the dura with interruption of the cortical vein can lead to acute neurological changes with clinical symptoms similar to those of symptomatic PLE.^3^ (3) Electrode tissue compatibility and immune factors or neurotoxicity of the implant: this was attributed to a fact that implant autopsy revealed a foreign body multinucleated giant cell‐type reaction present in all patients with DBS, and this reaction was present irrespective of the duration of implantation and may be a response to the polyurethane component of the electrodes' surface coat,^4^ but the study showed that the DBS leads did not contain any neurotoxins.^5^ (4) Short‐term inflammatory irritation: lead may stimulate an inflammatory tissue response, including microglial activation and astrogliosis, which may participate in inflammatory PLE.^6^ In addition, no studies have reported any relationship between gender, age, disease duration, score, comorbidities, surgical target, types of electrodes, number of implantations, whether microelectrode recording, or fibrin glue was used or not, and whether stimulation was turned on postoperatively. Therefore, prospective studies with large sample sizes are needed to clarify any relationship.

Taken together, these findings suggest that the etiology, susceptibility factors, and prognosis of this complication are still unclear. We conduct a systematic literature review and meta‐analysis study based on different viewpoints of this complication in different medical centers with the aim of clarifying the occurrence and development of this complication and identifying appropriate treatment strategies, so as to raise the academic attention to this complication.

## 2 METHODS

### 2.1 Search strategy and selection criteria

This systemic review was performed per the criteria outlined in the Preferred Reporting Items for Systematic Reviews and Meta‐Analyses (PRISMA) 2009 guidelines, and the PRISMA 2020 updated guideline statement.^7^, ^8^ This review was exempt from the requirements of an Institutional Review Board approval or informed patient consent. A literature search in English with no time restrictions was conducted on PubMed (MEDLINE) and Web of Science databases. The search string was built as follows: (edema) AND (DBS OR deep brain stimulation). The electronic database search was supplemented by a manual search of the reference lists. The reference list of all selected articles was independently screened to identify additional studies that may have been left out in the initial search.

The study question was formulated based on the Population, Intervention, Comparison, Outcome, and Study design (PICOS) strategy. Studies were included if they met the following criteria: Patients with Parkinson's disease who underwent DBS operation (Population), unilateral, or bilateral DBS (Intervention), none (Comparison), and brain edema, or symptomatic brain edema (Outcome). All study designs were eligible for inclusion. However, experimental studies, letters, comments, and editorials were excluded. Cross‐checking was done to avoid data duplication. All studies were evaluated for eligibility in the first screening phase based on a quick review of the title and the abstract. In the second screening phase, the full‐text articles were reviewed to evaluate eligibility criteria.

### 2.2 Data extraction

All articles were reviewed by three independent investigators. The investigators consulted among themselves in cases where there were differing opinions or consulted other experts if an agreement could not be reached. Each study contained the following features: first author, publication year, study type, sample size, imaging modality, the incidence of PLE cases, the incidence of symptomatic PLE cases, time duration to PLE onset postoperatively, time duration to symptomatic PLE onset postoperatively, results, and conclusions. Cross‐checking was further carried out after data extraction.

### 2.3 Quality assessment of included studies

The methodological quality of all studies was determined by one author, while two other authors did the discussion and verification to achieve consensus and establish an agreement with overall rating scores. The quality of the included studies was assessed by three independent investigators using the strengthening the Methodological Index for Non‐randomized Studies (MINORS) method whereby items were scored 0 if not reported, 1 if the reporting was inadequate, and 2 if the reporting was adequate. The global ideal score was 16 for noncomparative studies and 24 for comparative studies. The final assessment result was decided after the discussion. The maximum score was 16 since none of the studies included was a comparative study.

### 2.4 Evaluation of publication bias

The Peters’ test was performed to evaluate the publication bias. The Peters' test for the 10 studies was 0.763, indicating no significant publication bias. To further evaluate potential publication bias, we performed a sensitivity analysis. No obvious bias was revealed among the studies.

### 2.5 Statistical analysis

Meta‐analyses were conducted using R software 4.1.1 with Meta package 4.18–2 and the R package "Metaprop" for determining the proportion of PLE and symptomatic PLE. The results were presented as proportions at 95% confidence intervals (CIs). Forest plots were used to show the difference between the proportion of PLE and the symptomatic PLE after DBS operation. Heterogeneity among the studies was evaluated by *I*² and Cochrane's Q statistics. For *I*² < 40%, a fixed‐effects approach was used. The between‐study variance was determined by the random‐effects method.

## 3 RESULTS

### 3.1 Study selection

**FIGURE 1:**
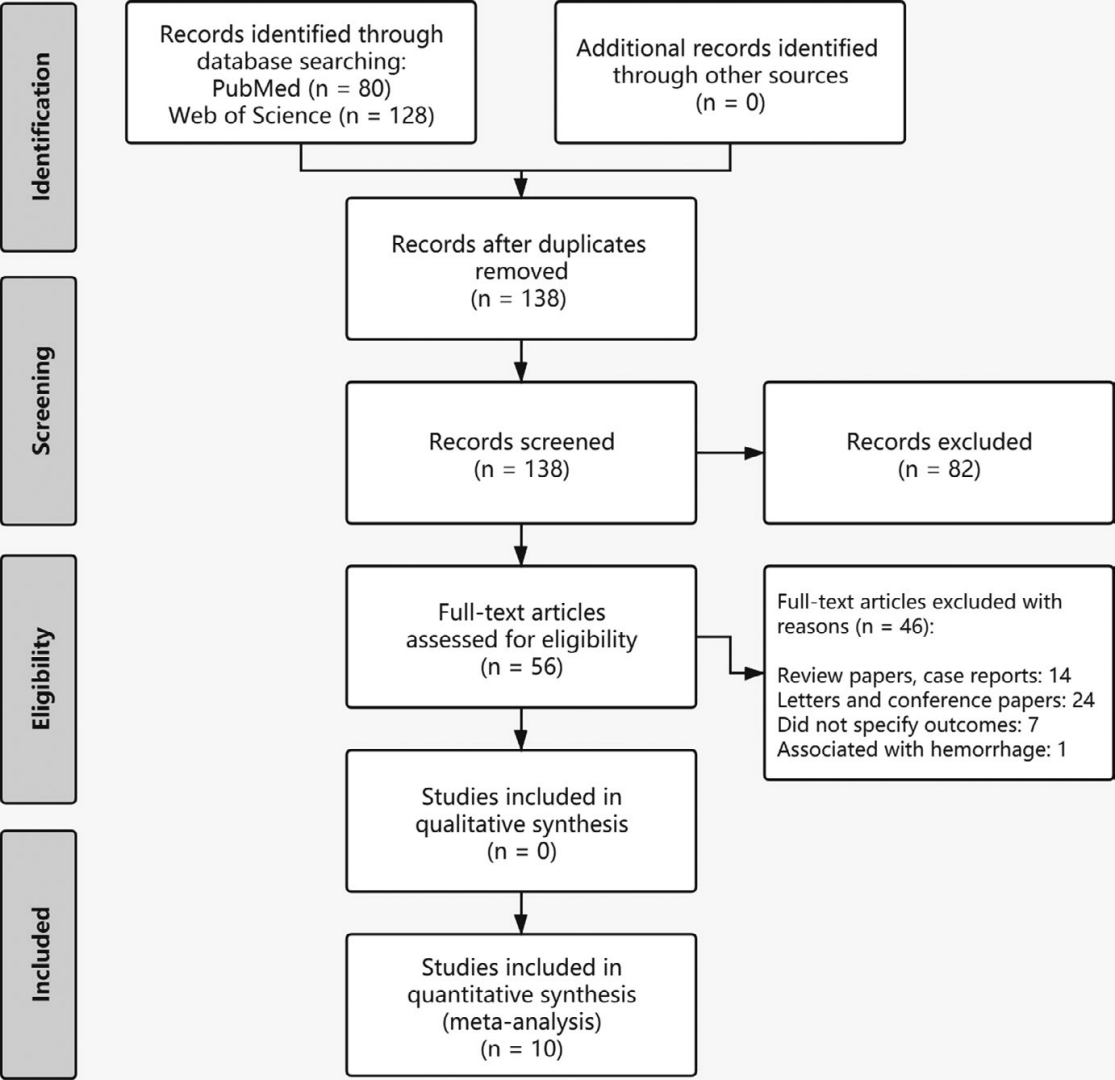
PRISMA flowchart showing systematic study selection

**FIGURE 2:**
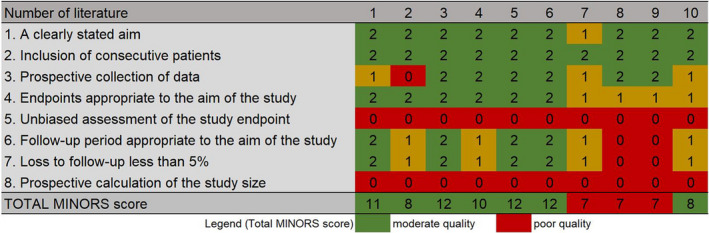
Study quality assessment (MINORS score)

The literature search yielded a total of 138 articles after removing duplicates. Eighty‐two articles were excluded after the first screening phase, leaving 56 articles that underwent full‐text review. Furthermore, an additional 46 articles were excluded after the second screening phase. Therefore, 10 articles reporting on peri‐lead cerebral edema were included in this study. The study selection process is represented in a PRISMA flowchart (Figure 1). Quality Assessment of included studies is represented in Figure 2.

### 3.2 Study characteristics

Characteristics of the articles included in the final literature review are presented in Table 1. The 10 studies were conducted between 2013 and 2021. Eight studies were retrospective, while two studies were prospective. Five of the 10 articles reported on PLE (Table 1, No.1–5), while all 10 reported on symptomatic PLE (Table 1, No.1–10). The 10 studies had a total patient number of 1354 (mean: 138.7; range: 13–260) who underwent DBS operation. According to seven of the articles, a total of 1609 electrodes were implanted, including 151 patients with unilateral lead implantation and 729 patients with bilateral lead implantation. Postoperative MRI scans were performed on 550 patients in six studies, while CT scans were performed on 815 patients in four studies. The implantation target areas included STN, GPI, VIM, and PSA. Peri‐lead cerebral edema was detected in 61 of 301 patients in five studies, while symptomatic PLE was detected in 51 of 1365 patients in 10 studies. Only four studies reported the follow‐up time (range: 10 days–10 weeks). On the other hand, only seven studies reported using microelectrode recording (MER) or multiple electrode trajectories. Although the studies provided a detailed account of chronic disease history, only two studies related the patient status to the chronic disease history.

**TABLE 1:** Characteristics of the articles included in the final literature review

No.	Author (Published year)	Study type	Ages	No. of patients (No. of leads)	Imaging modality (Imaging time postop)	No. of PLE (No. of leads)	No.of symptomatic PLE (No. of leads)	PLE onset postop	Symptomatic PLE onset postop	Comments/importance
1	Nolt et al.^21^ (2021)	Prospective	60.13	13 (15)	MRI (1 d, 2 w, 4 w, 6 w, 10 w)	11 (12)	0 (0)	1 d:4, 2 w:5, 4 w:2, 10 w:1	‐	Postoperative peri‐lead edema (PLE) is not rare, is often asymptomatic, and may resolve over many weeks.
2	Saitoh et al.^22^ (2019)	Retrospective	\	15 (\)	MRI (3−10 d)	6 (\)	0(0)	\	‐	PLE is not an uncommon phenomenon; 1 patient eosinophil count increased.
3	Whiting et al.^18^ (2018)	Prospective	64.1	102 (191)	MRI (2 m)	15 (17)	7 (\)	59.1 d	48.9 d	PLE may be more common than previously; no clear risk factors have been identified reported.
4	Englot et al.^23^ (2011)	Retrospective	62	133 (239)	MRI (0−13 d)	14 (15)	4 (4)	5.1 ± 0.9 d	4.5 d	PLE was typically unilateral, even in patients with simultaneous bilateral lead implants.
5	Ryu et al.^24^ (2004)	Retrospective	\	38 (38)	MRI (\)	15 (15)	0(0)	\	‐	PLE were detected only during the first 3 months after implantation in a significant number of patients.
6	Sharma et al.^25^ (2020)	Retrospective	\	260 (482)	CT (\)	‐	16 (20)	‐	5.8 d	Symptomatic PLE is an uncommon complication which typically resolves over time.
7	Fernandez‐Pajarín et al.^26^ (2017)	Retrospective	\	249 (\)	MRI (\)	‐	2 (\)	‐	\	The formation of intraparenchymal cysts seems to be the progression of PLE.
8	Fenoy et al.^27^ (2017)	retrospective	\	145 (281)	CT (\)	‐	5 (6)	‐	4.4 d	Acute instances of PLE may occur as early as POD 1 and can rapidly progress into profound deficits.
9	Nazzaro et al.^28^ (2017)	Retrospective	63.5	189 (363)	CT (\)	‐	10 (12)	‐	6.4 d	Symptomatic PLE is more common in re‐implantations compared with new implantations.
10	Kim et al.^6^ (2013)	Retrospective	56	221 (\)	CT (\)	‐	7 (10)	‐	4.4 d	Symptomatic PLE are thought to constitute a self‐limiting disorder requiring no additional treatment.

Abbreviations: d, day; m, month; w, week; “‐” represents not needed, “\” represents not described in the literature.

### 3.3 Main findings

In five studies, PLE was detected in 61 of 301 patients (incidence of 1.5%–4.7%). The incidence rate was reported as 30% in the retrospective studies and 50% in the prospective studies. However, in the 10 studies, symptomatic PLE was detected in 51 of 1365 patients (incidence of 0%–6.8%), with an incidence rate of 2.7% in the retrospective studies and 3.4% in the prospective studies. The duration of PLE ranged from one to 70 days, with an average of 31.6 days. However, the symptomatic PLE ranged from 4.4 to 48.9 days, with an average of 11.6 days. The included articles did not report any symptoms of long‐term neurological damage.

The imaging findings revealed that PLE mainly occurs as localized mechanical vasogenic cerebral edema, with a good prognosis and no damage to the brain parenchyma. Furthermore, PLE occurs as a delayed reaction with no cases reported on the first day after surgery. In addition, PLE can be located near the tip, in the subcortical region, or around the whole electrode. However, it is not reported which form is more common. Despite most DBS procedures being implanted with bilateral electrodes, PLE tends to be unilateral. The literature review did not reveal the laterality of edema occurrence and motor symptom laterality in patients with Parkinson's disease, but relevant studies show that the laterality of PLE is not related to the number of electrodes implanted, the order of bilateral implantation, or whether microelectrode recording is used or not.

### 3.4 Meta‐analysis

**FIGURE 3:**
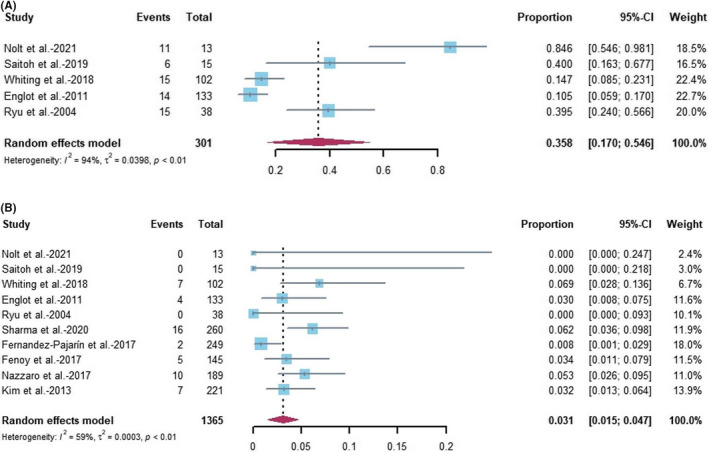
Forest plot of pooled estimate incidence of edema after DBS operation across all studies (A: Forest plot of studies on PLE; B: Forest plot of studies on symptomatic PLE). 95% CI, 95% confidence intervals; DBS, deep brain stimulation; PLE, peri‐lead edema

Ultimately, we analyzed the difference in the incidence of PLE and symptomatic PLE, according to the study methods and objectives of the included studies. For the incidence of PLE, five studies (three retrospective and two prospective case series) were included in the random‐effects model of single‐arm meta‐analysis with a total of 275 patients. Overall, the pooled PLE incidence was 35.8% (95%CI: 17.0%–54.6%; Figure 3A). Heterogeneity between studies was assessed using the *I* ^2^ index and Cochrane's *Q* test. The *I* ^2^ and *Q* statistics were 93.7% (95%CI: 88.2%–96.6%) and 66.32(*p* < 0.0001). The studies showed moderate to high heterogeneity.

For determination of the incidence of symptomatic PLE, 10 studies (eight retrospective and two prospective case series) were included in the random‐effects model of single‐arm meta‐analysis with a total of 1365 patients. Overall, the pooled incidence of symptomatic PLE was 3.1% (95%CI: 1.5%–4.7%) (Figure 3B). The *I* ^2^ and *Q* statistics were 59.1% (95%CI: 17.8%–79.6%) and 22.01(*p* < 0.01). The studies showed moderate heterogeneity.

**FIGURE 4:**
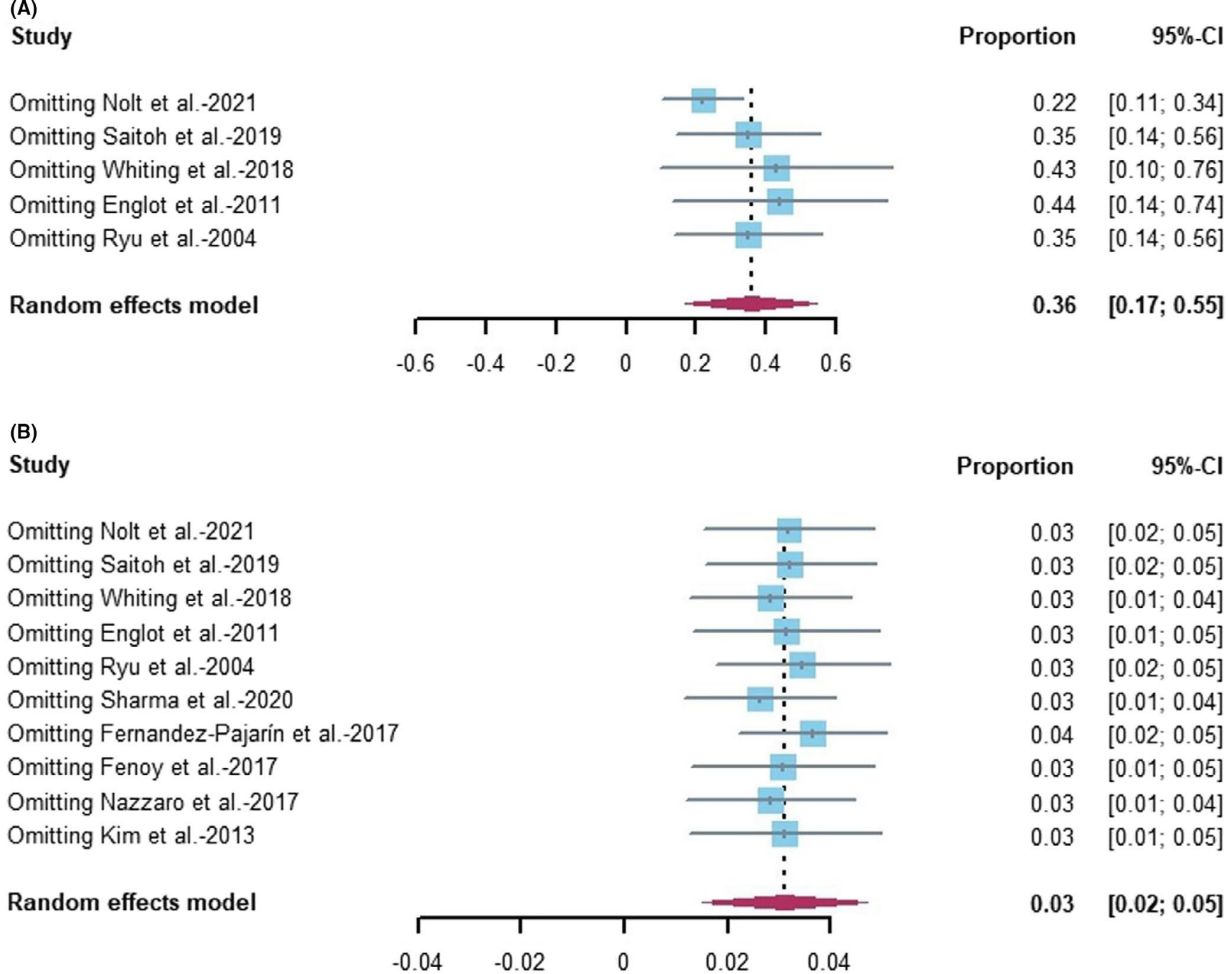
Sensitive analysis of estimate incidence for patients with edema after DBS operation (A: PLE B: symptomatic PLE). 95% CI, 95% confidence intervals; DBS, deep brain stimulation; PLE, peri‐lead edema

The steadiness and reliability of the meta‐analysis were analyzed by omitting one study at a time from the analysis. The results showed that no individual study affected the pooled incidence (Figure 4A,B). A subgroup analysis was not performed due to the few studies included. Further, the studies did not have comparator groups.

## 4 DISCUSSION

Cerebral edema occurs due to fluid accumulation in the intracellular and extracellular spaces of the brain due to extravasation from blood vessels. In the literature, different terms have been used for peri‐lead cerebral edema, including postoperative peri‐lead edema (PLE), postoperative peri‐electrode edema (PEE), idiopathic delayed‐onset edema (IDE), and dramatic radiographic abnormalities seen after electrode placement (DRAAEP) with contrast enhancement (CE). Here, we conducted a meta‐analysis and systematic literature review of clinical studies (Table 1) and case reports (Table 2) on PLE.

**TABLE 2:** Details of all published cases of symptomatic postoperative peri‐lead edema (PLE)

Author (Published Year)	Gender	Diagnosis (duration)		Chronic diseases	Improvement rate			Brain target (laterality)	Lead model	Edema laterality		Syndrome onset postop		Syndrome duration	Edema duration	DBS stimulation		Imaging modality	Symptoms	Intervention
Lefaucheur et al.^29^ (2013)	Male	PD (\)		\	55%			STN (bilateral)	Medtronic 3389	Right		10 d		3 w	3 w	Off		CT	Acute confusion with predominant behavioral troubles and headaches.	Oral corticosteroids
Jagid et al.^12^ (2015)	Male	PD (10 yr.)		\	56% (L) 62% (R)			STN (bilateral)	Medtronic 3389	Bilateral		4 m		9 m	14 m	On		CT/MRI	Severe decline in the cognitive abilities.	Steroid therapy
Arocho‐Quinones et al.^14^ (2016)	Male	PD (\)		\	\			STN (right)	\	Right		9 d		32 min	6 d	Off		CT/MRI	Seizure: left facial twitching and followed by disorientation.	Anticonvulsant medication
Gerard et al.^30^ (2016)	Male	PD (\)		\	78%			STN (bilateral)	Medtronic 3389	Left		7 d		1 w	4 m	\		MRI	Poor mentation, confusion, and expressive aphasia.	Cephalexin
Schoen et al.^31^ (2017)	Male	PD (8 yr.)		Colon cancer hypertension	62%			STN (bilateral)	Medtronic 3389	Left		33 h		1 w	8 d	Off		CT/MRI	Bilateral severe headache and nausea.	Dexamethasone
Staudt et al.^32^ (2018)	Male	ET (50 yr.)		‐	\			Vim (bilateral)	Vercise	Left		3 m		9 m	14 m	On		CT	Slurring of speech, mild disorientation, and gait imbalance.	Observation
Lee et al.^33^ (2019)	Male	PD (10 yr.)		\	\			STN (bilateral)	Medtronic 3387S	Left		6 h		3 d	6 d	Off		CT/MRI	Limited speech.	Dexamethasone
Fenoy et al.^34^ (2020)	Female	PD & ET (\)		\	\			Vim (bilateral)	Medtronic 3387	Left		1 d		40 d	3 m	Off		CT	Lethargic, global aphasia, plegic right upper extremity.	Dexamethasone/vancomycin and meropenem
Domino et al.^35^ (2021)	Male	CS (\)		\	\			Gpi (bilateral)	\	Left		18 d		2 w	1 m	\		CT	Headaches, altered mental status, difficulty speaking.	Dexamethasone and levetiracetam

Abbreviations: CS, Cockayne syndrome; ET, essential tremor; PD, Parkinson's disease; d, day; w, week; m, month; yr., year; “\” represents not described in the literature.

### 4.1 Causes and occurrence of postoperative peri‐lead edema

The patients did not present with fever after surgery, had the blood routine and inflammatory factors, within the normal range and had negative fungal and bacterial cultures. This shows that the patients did not have any infection. Although infections are common after DBS implantation, tracking infection along the DBS lead in brain parenchyma is uncommon. In most cases, PLE is self‐limiting, and the resolution without administration of any antibiotics suggests that PLE does not occur due to an infectious process.^9^, ^10^ Therefore, there is no sufficient evidence to support that an infection causes PLE.

Moreover, PLE may occur due to microhemorrhage or mechanical trauma to the brain tissue during the DBS lead implantation. Most imaging scans during target verification after DBS implantation do not show evidence of hemorrhage. However, implantation of the microelectrodes or macroelectrodes may cause local microhemorrhage and mechanical trauma to the brain, leading to impaired BBB and increased permeability resulting in “luxury perfusion.’’^9^ However, the "luxury perfusion" does not explain the confinement of the PLE around the electrode tip. In addition, PLE may be caused by the flow of CSF from the subarachnoid space into the brain parenchyma along the electrode puncture needle tract, thus accumulation after electrode implantation.

In literature, symptomatic PLE has also been attributed to venous infarction due to obstruction of the venous return.^11^ However, CT imaging of typical venous cerebral infarction shows irregular hypodense infarct foci in the cortex and subcortex, with irregular patchy species of high‐density hemorrhagic foci. In contrast, CT features of PLE showed a homogeneous hypodense shadow around the intracranial leads and under the cortex, which is somewhat different from the cortical arteriovenous infarction. Often, PLE has a delayed onset, whereas the clinical symptoms of cortical venous infarction are often (sub)acute. However, the cortical venous infarction does not explain the isolated cerebral edema confined to the electrode tip reported in some cases. Morishita et al.^11^ reported that high‐resolution, contrast‐enhanced MRI to delineate cerebral venous anatomy and improve the planning of the stereotactic trajectory could help avoid venous infarction. However, the literature review did not show a significant association between PLE and the planning of the surgical path.

Postoperative peri‐lead edema has also been associated with immune hypersensitivity reactions to the implant materials used (including DBS electrodes, extension leads, irrigation solutions, and fibrin sealant). Most reported PLE cases show a dramatic response to short‐term treatment with steroid therapy. Therefore, this response may be due to hypersensitivity or allergic reaction to the implanted material. Saitoh et al. reported a case of PLE with elevated eosinophils, but the patient underwent allergic reaction tests to the DBS materials after the occurrence of PLE showed no hypersensitivity reactions.^9^, ^12^ In addition, most cases of bilateral DBS implantation showed unilateral edema, which could not be inferred to immunogenicity.^9^

Possible neurotoxicity due to the DBS electrodes or leads may also cause PLE. A Medtronic product manual warns that polyurethane lead components may have neurotoxicity.^13^ Furthermore, the results of long‐term animal studies demonstrated the safety and biocompatibility of DBS leads made from polyurethane.^5^ Therefore, further studies are needed to show the correlation between the potential neurotoxic effects of DBS hardware and the acute and chronic reactions after surgery.

The irrigation solutions vary from center to center, including dilute betadine, dilute bacitracin solution,^9^ sterile water without betadine or bacitracin, saline solution with vancomycin,^14^ among others. Although these irrigation solutions have a risk of chemical cerebritis and edema, the literature denies that PLE is associated with lead irrigation. Cuba et al. used fibrin sealant in seven patients intraoperatively. They concluded that the occurrence of PLE was independent of the fibrin sealant.^15^ Fibrin sealant may minimize CSF loss during surgery. However, the use of fibrin sealant is not widespread. Furthermore, it has not been demonstrated whether fibrin sealant is associated with PLE. Overall, the involvement of allergic reactions and immune response to PLE complications has not been fully confirmed. Therefore, more studies are needed to elucidate the underlying mechanisms.

Other possible causes of PLE include acute inflammatory reactions after DBS implantation.^6^ Biran et al.^16^ reported that the method of anchoring silicon microelectrode arrays to the skull significantly led to shearing and/or compression of the adjacent tissue. This could arise from a stiffness mismatch between the implant and brain tissue caused by the relative motion between the skull and the brain parenchyma. Most studies demonstrate spontaneous healing of PLE without any treatment. However, studies do not explain the unilaterality of PLE or confinement around the electrode tips. A prospective study conducted by Borellini et al.^17^ reported 100% incidence of PLE in the 19 patients included. However, this study was excluded from our meta‐analysis due to the small sample size and the occurrence of peri‐electrode hemorrhage in six patients.

It is not known whether PLE is related to changes in the vascular structure due to chronic diseases such as hypertension and diabetes mellitus, and to spasm of small blood vessels in the brain due to implant irritation, causing exudation and edema. However, according to the study, this complication was not associated with the presence of vascular disease, hypertension, diabetes, or the use of anticoagulants/antiplatelet agents, but due to the small sample size and the fact that the MRI scan was performed only 6 weeks after surgery, no accurate conclusions can be drawn.^18^ So, it remains to be investigated whether comorbidities are related to PLE and whether the related changes can be alleviated by drugs to improve microcirculation and electrode surface irrigation.

### 4.2 Symptoms, course, prognosis, and treatment of postoperative peri‐lead edema

The meta‐analysis and literature review showed that the incidence of PLE was 35.8%, while the incidence of symptomatic PLE was 3.1%, accounting for an overall incidence of 8.7% of the PLE cases. This finding suggests that the onset of PLE may be a slow process with a low proportion of neuropsychiatric symptoms. Patients present with varying clinical symptoms, including headache, nausea, confusion, a decline in cognitive function, mood changes, aphasia, disorientation, gait instability, muscle strength loss/paralysis, and epilepsy. Studies do not show any relationship between the development of symptoms and PLE. Therefore, the association of neurological symptoms with the edema volume and the involved area requires further investigation. However, studies show a relationship between the development of epilepsy and PLE. A retrospective study including 161 patients with DBS implantation reported that seven patients had epilepsy symptoms after surgery (4.3%). Postoperative edema, hemorrhage, or ischemia increases the relative risk of postoperative seizures by 30‐ to 50‐fold. However, long‐term anticonvulsant therapy is not likely required because these patients do not develop chronic epilepsy.^19^

The PLE complication is often self‐limiting. Patients with asymptomatic PLE tend to recover spontaneously, with recovery time ranging from 1 to 70 days, with a mean of 31.6 days. Patients who develop neuropsychiatric symptoms recover fully without any neurological damage upon administration of treatment, with a duration of symptoms ranging from 4.4 to 48.9 days, with a mean of 11.63 days. However, the formation of cysts has been reported in cases of cerebral edema left untreated. Jagid et al.^12^ reported a case of a patient who developed bilateral symptomatic PLE four months after DBS, which worsened five months postoperatively. In this patient, no specific treatment was given during the entire period. A review of the images done nine months postoperatively revealed that the edema had developed into a large nonenhancing cystic cavity resembling encephalomalacia. The patient was then treated with steroids, and he recovered 13 months after surgery. Therefore, although most cases of symptomatic PLE heal without treatment, surgeons need to be alert to worsening patient's symptoms due to worsening cerebral edema or the development of cysts.

Patients with simple asymptomatic PLE can recover without specific treatment; however, they should be clinically monitored to assess PLE progression and emergence of clinical symptoms. Patients presenting with seizures should be administered with appropriate medications. PLE patients presenting with neuropsychiatric dysfunction should be urgently treated. Dehydration treatment alone did not have a significant effect. Most of the treatment regimens that have been reported in literature are hormonal (steroids 1 mg/kg/day and dexamethasone 4 mg q.6h). Mechanistically, hormones can inhibit inflammatory responses, reduce microvascular permeability, stabilize cell membranes, restore sodium pump functions, improve mitochondrial functions, prevent or attenuate free radical‐induced lipid peroxidation, and are effective in inflammation‐induced interstitial cerebral edema. Therefore, hormonal therapy is effective for edema of vascular origin. Also, it was not necessary to remove the electrodes and extension leads to avoid reoperation, which would have been traumatic for the patient.

### 4.3 Reasons for different views

This review reveals that PLE is not an uncommon complication. The improved compatibility of the DBS hardware with MRI imaging has greatly improved the detection rate of PLE in recent years. The difference between the previously reported low occurrence of PLE and the findings of this review may be explained by the following: (1) the occurrence of PLE is characterized by a delayed onset and a low detection rate within the 1‐day postoperative review window; (2) the detection of PLE is incidental as patients usually have no obvious clinical symptoms; (3) most doctors detect PLE only after patients show clinical symptoms, which explains why PLE is usually detected within a few weeks after surgery, consistent with delayed onset of symptomatic PLE; (4) An assumption that the PLE occurred when it was detected by imaging may have overlooked the fact that PLE may have been present longer than recorded; (5) in the past, interference from electrode and extension lead made it challenging to detect microscopic brain edema on CT. In addition, infection is a common complication of DBS.^20^ Detection of a large area of low‐density shadow on CT may be considered as an infection. However, PLE after DBS operation may also present similarly in CT imaging, and this may further explain the underreporting of PLE.

## 5 LIMITATIONS

This study is associated with various limitations. For ethical and feasibility reasons, the currently available studies, both prospective and retrospective, did not have control groups; therefore, we do not exclude the potential impact of some confounding factors on our meta‐analysis. To minimize the effects of confounding factors, we used the meta‐analysis guidelines and MINORS scoring methods to rigorously assess the included studies. Sources of heterogeneity were not explored in this study as the number of included studies was limited. Since demographic data were not obtained, the relationship between some independent factors, such as age, presence of comorbidities, examination method, and follow‐up time with incidences of postoperative PLE were not determined. The single‐arm meta‐analysis was inherently less stable than the 2‐arm, which was one of the reasons for the high heterogeneity. In addition, published studies have variances regarding time intervals for imaging after operation; therefore, the time process of PLE was simply reported as the time of initial observation, while the actual onset was at some unknown time point before that, therefore, the course of PLE may be longer than described, which may have had an influence on our results.

## 6 CONCLUSIONS

Deep brain stimulation does not cause major structural damage to the brain; however, several complications can occur during clinical treatment. Based on recent findings from different neurosurgical centers, PLE is not a rare complication as previously thought. We established that the incidence of asymptomatic PLE was 35.8%, while that of symptomatic PLE was 3.1%. Their etiologies may be associated with infection, mechanical damage to the punctures lead, accumulation of CSF to electrode puncture needle tract, venous infarction, electrode tissue compatibility and immune factors, neurotoxicity of the implant, as well as short‐term inflammatory irritation. It has not been established which factors play a major role in the etiology of PLE. Asymptomatic PLE is self‐limiting. Patients with clinical symptoms might be treated with dehydration and hormonal therapy to achieve better prognostic outcomes without neurological sequelae.

## CONFLICT OF INTERESTS

The authors have no conflicts of interest to declare that are relevant to the content of this article.

## AUTHOR CONTRIBUTIONS

Yu Tian and Jiaming Wang contribute equally. Yu Tian and Jiaming Wang performed the data analyses and wrote the manuscript. Lei Jiang helped for giving the literature review and quality assessment of included studies. Zhaohai Feng helped for data extraction and quality assessment of included studies. Xin Shi contributed to the conception of the study, performed the operation, and gave the manuscript preparation. Yujun Hao helped to perform the analysis with constructive discussions.

## Data Availability

The data that support the findings of this study are available from the corresponding author upon reasonable request.

## References

[1] Chan DT , Zhu XL , Yeung JH , et al. Complications of deep brain stimulation: a collective review. Asian J Surg. 2009;32(4):258‐263. doi:10.1016/s1015-9584(09)60404-8 19892631

[2] Spataro L , Dilgen J , Retterer S , et al. Dexamethasone treatment reduces astroglia responses to inserted neuroprosthetic devices in rat neocortex. Exp Neurol. 2005;194(2):289‐300. doi:10.1016/j.expneurol.2004.08.037 16022859

[3] Binder DK , Rau GM , Starr PA . Risk factors for hemorrhage during microelectrode‐guided deep brain stimulator implantation for movement disorders. Neurosurgery. 2005;56(4):722‐732; discussion 722‐32. doi:10.1227/01.neu.0000156473.57196.7e 15792511

[4] Moss J , Ryder T , Aziz TZ , Graeber MB , Bain PG . Electron microscopy of tissue adherent to explanted electrodes in dystonia and Parkinson's disease. Brain. 2004;127(Pt 12):2755‐2763. doi:10.1093/brain/awh292 15329356

[5] Hooper S , Cameron T . Neurotoxicity screening test for deep brain stimulation leads. J Biomater Sci Polym Ed. 2007;18(10):1309‐1320. doi:10.1163/156856207782177873 17939888

[6] Kim JW , Hwang JH , Kim IK , et al. Acute brain reaction to DBS electrodes after deep brain stimulation: chronological observation. Acta Neurochir (Wien). 2013;155(12):2365‐2371; discussion 2371. doi:10.1007/s00701-013-1853-3 24009047

[7] Moher D , Liberati A , Tetzlaff J , Altman DG . Preferred reporting items for systematic reviews and meta‐analyses: the PRISMA statement. Ann Intern Med. 2009;151(4):264‐269, w64. doi:10.7326/0003-4819-151-4-200908180-00135 19622511

[8] Page MJ , McKenzie JE , Bossuyt PM , et al. The PRISMA 2020 statement: an updated guideline for reporting systematic reviews. Int J Surg. 2021;88:1‐9. doi:10.1016/j.ijsu.2021.105906 33789826

[9] Deogaonkar M , Nazzaro JM , Machado A , Rezai A . Transient, symptomatic, post‐operative, non‐infectious hypodensity around the deep brain stimulation (DBS) electrode. J Clin Neurosci. 2011;18(7):910‐915. doi:10.1016/j.jocn.2010.11.020 21571534

[10] Merello M , Cammarota A , Leiguarda R , Pikielny R . Delayed intracerebral electrode infection after bilateral STN implantation for Parkinson's disease. Case report. Mov Disord. 2001;16(1):168‐170. doi:10.1002/1531-8257(200101)16:1<168::aid-mds1032>3.0.co;2-n 11215583

[11] Morishita T , Okun MS , Burdick A , Jacobson CE , Foote KD . Cerebral venous infarction: a potentially avoidable complication of deep brain stimulation surgery. Neuromodulation. 2013;16(5):407‐413. discussion 413. doi:10.1111/ner.12052 23738501 PMC3772976

[12] Jagid J , Madhavan K , Bregy A , et al. Deep brain stimulation complicated by bilateral large cystic cavitation around the leads in a patient with Parkinson's disease. BMJ Case Rep. 2015;2015:bcr2015211470. doi:10.1136/bcr-2015-211470 PMC4612520 26475878

[13] Medtronic I . Lead Kit for Deep Brain Stimulation Implant Manual. Medtronic Inc; 2008.

[14] Arocho‐Quinones EV , Pahapill PA . Non‐infectious peri‐electrode edema and contrast enhancement following deep brain stimulation surgery. Neuromodulation. 2016;19(8):872‐876. doi:10.1111/ner.12432 27098925

[15] de Cuba CM , Albanese A , Antonini A , et al. Idiopathic delayed‐onset edema surrounding deep brain stimulation leads: Insights from a case series and systematic literature review. Parkinsonism Relat Disord. 2016;32:108‐115. doi:10.1016/j.parkreldis.2016.09.007 27622967

[16] Biran R , Martin DC , Tresco PA . The brain tissue response to implanted silicon microelectrode arrays is increased when the device is tethered to the skull. J Biomed Mater Res A. 2007;82(1):169‐178. doi:10.1002/jbm.a.31138 17266019

[17] Borellini L , Ardolino G , Carrabba G , et al. Peri‐lead edema after deep brain stimulation surgery for Parkinson's disease: a prospective magnetic resonance imaging study. Eur J Neurol. 2019;26(3):533‐539. doi:10.1111/ene.13852 30358915

[18] Whiting AC , Catapano JS , Walker CT , Godzik J , Lambert M , Ponce FA . Peri‐lead edema after deep brain stimulation surgery: a poorly understood but frequent complication. World Neurosurg. 2018;124:e340–e345. doi:10.1016/j.wneu.2018.12.092 30594699

[19] Pouratian N , Reames DL , Frysinger R , Elias WJ . Comprehensive analysis of risk factors for seizures after deep brain stimulation surgery. Clinical article. J Neurosurg. 2011;115(2):310‐315. doi:10.3171/2011.4.Jns102075 21548744

[20] Doshi PK , Rai N , Das D . Surgical and hardware complications of deep brain stimulation‐a single surgeon experience of 519 cases over 20 years. Neuromodulation. 2021. Epub ahead of print. doi:10.1111/ner.13360 33496063

[21] Nolt MJ , Polasani RS , Masnyk TW , Rezak M , Rosenow JM . Prospective evaluation of the time course of white matter edema associated with implanted deep brain stimulation electrodes. Stereotact Funct Neurosurg. 2021;99(3):203‐206. doi:10.1159/000511115 33221795

[22] Saitoh T , Enatsu R , Mikami T , et al. Peri‐electrode edema after deep brain stimulation. J Clin Neurosci. 2019;59:29‐31. doi:10.1016/j.jocn.2018.11.026 30472347

[23] Englot DJ , Glastonbury CM , Larson PS . Abnormal T2‐weighted MRI signal surrounding leads in a subset of deep brain stimulation patients. Stereotact Funct Neurosurg. 2011;89(5):311‐317. doi:10.1159/000329365 21894061

[24] Ryu SI , Romanelli P , Heit G . Asymptomatic transient MRI signal changes after unilateral deep brain stimulation electrode implantation for movement disorder. Stereotact Funct Neurosurg. 2004;82(2–3):65‐69. doi:10.1159/000077402 15305076

[25] Sharma VD , Lyons KE , Nazzaro JM , Pahwa R . Does post‐operative symptomatic lead edema associated with subthalamic DBS implantation impact long‐term clinical outcomes? J Neurol Sci. 2020;410:116647. doi:10.1016/j.jns.2019.116647 31901593

[26] Fernandez‐Pajarin G , Sesar A , Ares B , et al. Delayed complications of deep brain stimulation: 16‐year experience in 249 patients. Acta Neurochir (Wien). 2017;159(9):1713‐1719. doi:10.1007/s00701-017-3252-7 28646465

[27] Fenoy AJ , Villarreal SJ , Schiess MC . Acute and subacute presentations of cerebral edema following deep brain stimulation lead implantation. Stereotact Funct Neurosurg. 2017;95(2):86‐92. doi:10.1159/000454892 28208150

[28] Nazzaro JM , Pahwa R , Lyons KE . Symptomatic, non‐infectious, non‐hemorrhagic edema after subthalamic nucleus deep brain stimulation surgery for Parkinson's disease. J Neurol Sci. 2017;383:42‐46. doi:10.1016/j.jns.2017.10.003 29246619

[29] Lefaucheur R , Derrey S , Borden A , et al. Post‐operative edema surrounding the electrode: an unusual complication of deep brain stimulation. Brain Stimul. 2013;6(3):459‐460. doi:10.1016/j.brs.2012.05.012 22743074

[30] Gerard CS , Metman LV , Pal G , Karl J , Sani S . Severe, symptomatic, self‐limited unilateral dbs lead edema following bilateral subthalamic nucleus implantation: case report and review of the literature. Neurologist. 2016;21(4):58‐60. doi:10.1097/NRL.0000000000000082 27348140

[31] Schoen NB , Jermakowicz WJ , Luca CC , Jagid JR . Acute symptomatic peri‐lead edema 33 hours after deep brain stimulation surgery: a case report. J Med Case Rep. 2017;11(1):103. doi:10.1186/s13256-017-1275-6 28407815 PMC5391613

[32] Staudt MD , MacDougall KW . Spontaneous regression of an intraparenchymal cyst following deep brain stimulator electrode implantation: case report and literature review. World Neurosurg. 2018;117:249‐254. doi:10.1016/j.wneu.2018.06.115 29940379

[33] Lee JJ , Daniels B , Austerman RJ , Dalm BD . Symptomatic, left‐sided deep brain stimulation lead edema 6 h after bilateral subthalamic nucleus lead placement. Surg Neurol Int. 2019;10:68. doi:10.25259/sni-125-2019 31528406 PMC6744830

[34] Fenoy AJ , Conner CR , Withrow JS , Hocher AW . Case report of hyperacute edema and cavitation following deep brain stimulation lead implantation. Surg Neurol Int. 2020;11:259. doi:10.25259/SNI_527_2019 33024597 PMC7533082

[35] Domino JS , Lundy P , Kaufman CB . Fulminant non‐infectious peri‐electrode edema after deep brain stimulation system implantation in a pediatric patient. Childs Nerv Syst. 2021. doi:10.1007/s00381-021-05224-6 34057621

